# Compositional and functional differences of the vaginal microbiota of women with and without cervical dysplasia

**DOI:** 10.1038/s41598-024-61942-2

**Published:** 2024-05-16

**Authors:** Johanna Norenhag, Gabriella Edfeldt, Karin Stålberg, Fabricio Garcia, Luisa Warchavchik Hugerth, Lars Engstrand, Emma Fransson, Juan Du, Ina Schuppe-Koistinen, Matts Olovsson

**Affiliations:** 1 Department of Women’s and Children’s Health, Uppsala University, Uppsala, Sweden; 2 Department of Microbiology, Tumor and Cell Biology (MTC), Centre for Translational Microbiome Research, Karolinska Institutet, Stockholm, Sweden; 3 Department of Medical Biochemistry and Microbiology, Science for Life Laboratory, Uppsala University, Uppsala, Sweden

**Keywords:** Computational biology and bioinformatics, Medical research

## Abstract

Alterations in the vaginal microbiota, including both species composition and functional pathways, have been associated with HPV infection and progression of dysplasia to cervical cancer. To further explore this, shotgun metagenomic sequencing was used to taxonomically and functionally characterize the vaginal microbiota of women with and without cervical dysplasia. Women with histologically verified dysplasia (n = 177; low grade dysplasia (LSIL) n = 81, high-grade dysplasia (HSIL) n = 94, cancer n = 2) were compared with healthy controls recruited from the cervical screening programme (n = 177). Women with dysplasia had a higher vaginal microbial diversity, and higher abundances of *Gardnerella vaginalis, Aerococcus christensenii, Peptoniphilus lacrimalis* and *Fannyhessea vaginae*, while healthy controls had higher relative abundance of *Lactobacillus crispatus*. Genes involved in e.g. nucleotide biosynthesis and peptidoglycan biosynthesis were more abundant in women with dysplasia. Healthy controls showed higher abundance of genes important for e.g. amino acid biosynthesis, (especially L-lysine) and sugar degradation. These findings suggest that the microbiota may have a role in creating a pro-oncogenic environment in women with dysplasia. Its role and potential interactions with other components in the microenvironment deserve further exploration.

## Introduction

Human papillomavirus (HPV) is one of the world’s most common sexually transmitted infections^1^. Most HPV infections resolve within a few months to years^2^. A persistent infection with HPV can lead to cervical cancer, the fourth most common cancer among women globally^3,4^. Known risk factors for acquiring an HPV infection and developing cervical cancer include smoking, multiple sex partners and immune deficiency^5^.

To understand the progression from HPV infection to cervical cancer, several studies have explored the role of the vaginal microbiota. A healthy vaginal microbiota consists mainly of *Lactobacillus* species that help protect against pathogens by producing bacteriocins, biosurfactants and lactic acid^6–8^. A predominance of non-*Lactobacillus* species, as in bacterial vaginosis (BV), has been associated with a higher prevalence of HPV infection, cervical dysplasia and cancer^9^. Certain bacteria, such as the genera *Atopobium*, *Gardnerella* and *Sneathia,* have been proposed as microbial markers for HPV infection and the dysplastic progression to cervical cancer^9–11^. However, the molecular mechanisms of their contribution are still unexplored.

A more active role of the vaginal microbiota has been suggested as several bacterial species seem to be associated with both persistence and remission of HPV infection and dysplasia^10,12^. Recent mechanistic in vitro studies also show an inflammatory response and tumour promoting effect on cervical epithelia in the presence of bacteria such as *Peptoniphilus lacrimalis* and *Fusobacterium nucleatum *^13^. This is well in line with the addition of polymorphic microbiota as one of the enabling characteristics to the hallmarks of cancer^14^.

Previous studies on the vaginal microbiota in women with dysplasia have primarily used 16S rRNA gene sequencing, linking microbial compositions with HPV-related disease. The taxonomic resolution of 16S is often limited to genus level and restricted to bacteria. Shotgun metagenomic sequencing offers a higher taxonomic resolution and enables studying all the microbes in a sample, including bacteria, fungi, protozoa and viruses. This allows both for more comprehensive information on the microbial composition as well as information on its possible functional properties. Shotgun metagenomic analysis may therefore contribute to an increased understanding of what role the microbiota has for acquiring or maintaining an HPV infection and for the development of cervical cancer^15,16^. In this study we used metagenomic sequencing data to taxonomically and functionally compare the vaginal microbiota of women with cervical dysplasia with healthy controls.

## Results

### Study population

Cervico-vaginal samples for microbiota analysis were collected from a total of 354 women. Women with histologically verified dysplasia (n = 177; low grade dysplasia/ low-grade squamous intra-epithelial lesion (LSIL) n = 81, high-grade dysplasia/ high-grade squamous intra-epithelial lesion (HSIL) n = 94, cancer (ca) n = 2) were compared with healthy age-matched controls recruited from the cervical screening programme (n = 177).

A short health questionnaire at time of sampling revealed no difference between women with dysplasia and healthy controls with regards to time since last menstrual bleeding, antibiotic treatment, sexual intercourse during the past 24 h, and current gynaecological symptoms (Table 1, Table S1). When comparing women with low grade dysplasia (LSIL) with women with high-grade dysplasia or cancer (HSIL/Ca), women with HSIL/Ca more often reported vaginal discharge (Table 1).

**Table 1:** Demographic and clinical characteristics of the study participants.

	HC (n = 177)	Dysplasia (= 177)	*p*-value	LSIL (n = 81)	HSIL/ca (n = 96)	*p*-value	CIN2 (n = 23)	CIN3 (n = 45)	*p*-value
Age			0.964			0.269			0.692
Mean (SD)	35.3 (10.5)	35.2 (10.5)		36.5 (11.5)	34.1 (9.54)		34.9 (10.3)	35.5 (9.88)	
Median (range)	33 (23–68)	33 (21–70)		34 (21–70)	32 (22–62)		35 (22–62)	33 (22–62)	
Vaginal intercourse < 24 h before sampling			0.28			0.464			1
Yes	18 (10.2%)	11 (6.2%)		7 (8.6%)	4 (4.2%)		1 (4.3%)	2 (4.4%)	
No	148 (83.6%)	150 (84.7%)		71 (87.7%)	79 (82.3%)		18 (78.3%)	37 (82.2%)	
Missing	11 (6.2%)	16 (9%)		3 (3.7%)	13 (13.5%)		4 (17.4%)	6 (13.3%)	
Antibiotics within 3 months before sampling			0.796			0.415			0.795
Yes	9 (5.1%)	11 (6.2%)		7 (8.6%)	4 (4.2%)		2 (8.7%)	2 (4.4%)	
No	156 (88.1%)	152 (85.9%)		70 (86.4%)	82 (85.4%)		17 (73.9%)	39 (86.7%)	
Missing	12 (6.8%)	14 (7.9%)		4 (4.9%)	10 (10.4%)		4 (17.4%)	4 (8.9%)	
Regular menstrual bleedings			0.0731			0.674			1
Yes	122 (68.9%)	99 (55.9%)		44 (54.3%)	55 (57.3%)		12 (52.2%)	26 (57.8%)	
No	48 (27.1%)	61 (34.5%)		30 (37%)	31 (32.3%)		7 (30.4%)	15 (33.3%)	
Missing	7 (4%)	17 (9.6%)		7 (8.6%)	10 (10.4%)		4 (17.4%)	4 (8.9%)	
Weeks since last menstrual bleeding			0.401			0.238			0.275
1 week	31 (17.5%)	24 (13.6%)		11 (13.6%)	13 (13.5%)		5 (21.7%)	3 (6.7%)	
2 weeks	37 (20.9%)	30 (16.9%)		18 (22.2%)	12 (12.5%)		2 (8.7%)	8 (17.8%)	
3 weeks	32 (18.1%)	24 (13.6%)		8 (9.9%)	16 (16.7%)		4 (17.4%)	9 (20%)	
4 weeks	22 (12.4%)	21 (11.9%)		7 (8.6%)	14 (14.6%)		1 (4.3%)	6 (13.3%)	
No menstrual bleeding	48 (27.1%)	61 (34.5%)		30 (37%)	31 (32.3%)		7 (30.4%)	15 (33.3%)	
Missing	7 (4%)	17 (9.6%)		7 (8.6%)	10 (10.4%)		4 (17.4%)	4 (8.9%)	
Vaginal discharge < 24 h before sampling			0.636			0.0273			0.552
Yes	8 (4.5%)	11 (6.2%)		1 (1.2%)	10 (10.4%)		4 (17.4%)	4 (8.9%)	
No	152 (85.9%)	149 (84.2%)		72 (88.9%)	77 (80.2%)		17 (73.9%)	36 (80%)	
Missing	17 (9.6%)	17 (9.6%)		8 (9.9%)	9 (9.4%)		2 (8.7%)	5 (11.1%)	

Clinical information from questionnaires with study participants grouped according to histopathological diagnosis; Healthy controls (HC), dysplasia of any type (Dysplasia), low-grade squamous intra-epithelial lesion (LSIL), high-grade squamous intra-epithelial lesion (HSIL), cervical intra-epithelial neoplasia 2 (CIN2), cervical intra-epithelial neoplasia 3 (CIN3). *p*-values were calculated using Kruskal–Wallis or Chi-Square test.

### Microbial diversity, taxonomic composition and functional pathways differ between women with dysplasia and healthy controls

**Figure 1:**
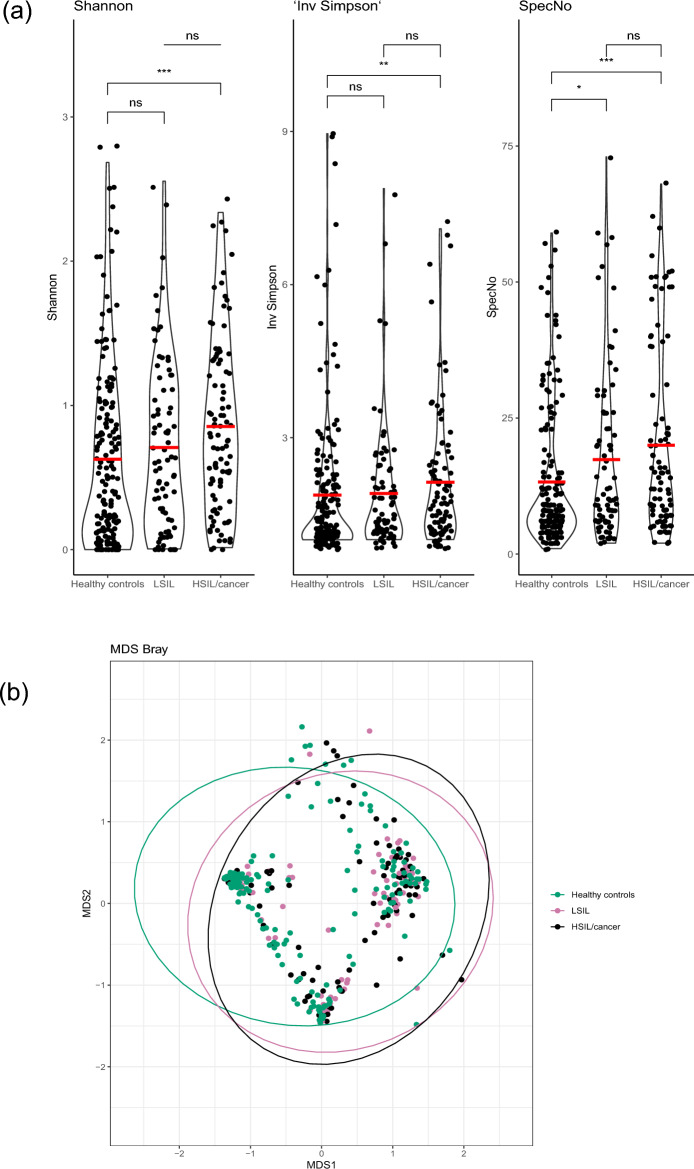
Microbial diversity according to histopathological diagnosis. (**a**) Alpha diversity measures (Shannon, inverse Simpson’s and species count) according to histopathological diagnosis; healthy controls (HC), low-grade squamous intra-epithelial lesion (LSIL), and high-grade squamous intra-epithelial lesion (HSIL)/cancer. A significantly higher alpha-diversity was observed for women with HSIL compared with healthy controls. Women with LSIL showed higher species numbers compared with healthy controls. There were no differences between LSIL and HSIL/ca. (**b**) Microbial beta-diversity in Healthy controls, LSIL and HSIL. The healthy controls cluster separately from the two dysplasia groups (LSIL and HSIL/ca) in a two-dimensional non-metric multidimensional scaling (NMDS) of distances between samples. Permutational multivariate analysis of variance (PERMANOVA) comparing all three groups shows a significantly different microbiome composition with *p*-value < 0.001. Separate analysis comparing LSIL and HSIL did not show any difference (*p* > 0.05), hence the main difference is between HC and LSIL/HSIL.

The vaginal microbiota alpha-diversity was higher among women with dysplasia compared with healthy controls (*p*-values < 0.002) (Fig. 1a). The beta diversity also confirmed a difference in taxonomic composition between women with dysplasia and healthy controls (permanova *p*-value < 0.001, R2 0.022) (Fig. 1b).

**Figure 2:**
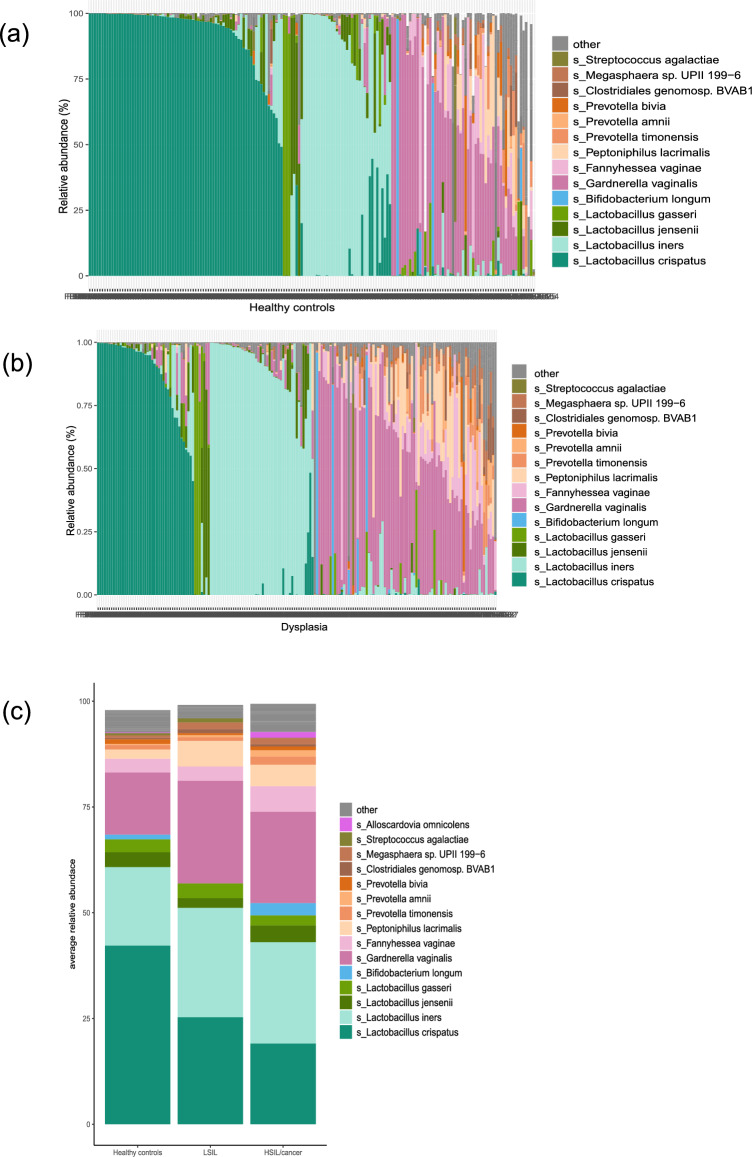
Microbial composition in healthy controls and women with dysplasia. Relative abundance per sample, with the 14 most common species in colour for (**a**) healthy controls (n = 177) and (**b**) women with dysplasia (n = 177). Figure (**c**) shows the average relative abundance according to histopathological diagnosis; healthy controls (HC), low-grade squamous intra-epithelial lesion (LSIL), and high-grade squamous intra-epithelial lesion (HSIL)/cancer.

**Figure 3:**
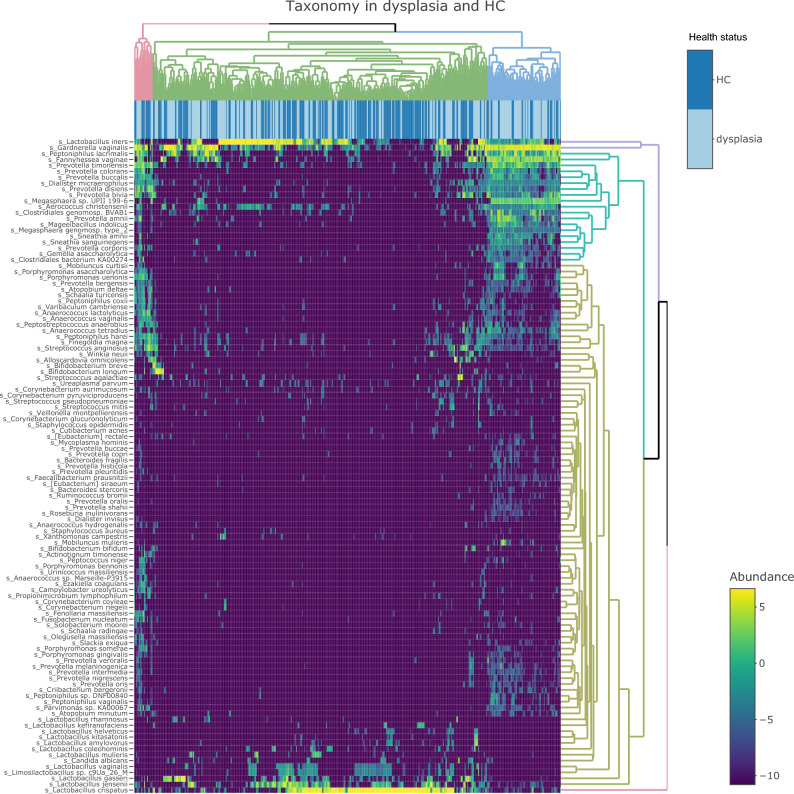
Heatmap showing the microbial taxonomic abundance in healthy controls and women with dysplasia. Heatmap of the relative abundance of microbial taxa in healthy controls (HC) marked in dark blue bars on top of the heatmap, and women with dysplasia marked in light blue. Clusters of samples with similar microbial composition emerging from the data are highlighted in different colours on top of the heatmap. Taxa that clusters due to similar patterns in the data are highlighted in different colours on the right side of the heatmap. Relative abundance data were log2 normalized for better visualization.

Differential abundance analysis showed that *Gardnerella vaginalis, Aerococcus christensenii, Peptoniphilus lacrimalis* and *Fannyhessae vaginae* were more abundant in women with dysplasia while *Lactobacillus crispatus* was more abundant in healthy controls (Table S2, Figs. 2 and 3).

The samples were categorized into community state types (CSTs) based on the dominant species in their bacterial composition as described^17^, with four CSTs dominated by *Lactobacillus* species; CST-I (dominated by *L. crispatus*), CST-II (dominated by *L. gasseri*), CST-III (dominated by *L.iners*) and CST-V (dominated by *L. jensenii*), while CST-IV is composed mainly by non-lactobacilli species, with no dominant species and can be sub grouped further based on the microbial composition. For the healthy controls in our study CST I (42.4%) was the most common, followed by CST IV (31%) and CST III (24.3%). The most common CST among women with dysplasia was CST IV (44.6%), followed by CST III (26.0%) and CST I (24.3%) (*p*-value < 0.005) (Table 2, Table S3).

**Table 2:** Microbial compositions based on community state types (CST’s) according to study participant’s histopathological diagnosis.

Number of study participants n, (%)	CST I	CST II	CST III	CST IV-A	CST IV-B	CST IV-C	CSTV	*p*-value
HC (n = 177)	75 (42.4)	4 (2.3)	36 (20.3)	2 (1.1)	42 (23.7)	11(6.2)	7 (4.0)	0.00467
Dysplasia (n = 177)	43 (24.3)	5 (2.8)	46 (26.0)	2 (1.1)	70 (39.5)	7 (4.0)	4 (2.3)	
LSIL (n = 81)	24 (29.6)	2 (2.5)	21 (25.9)	1 (1.2)	31 (38.3)	1 (1.2)	1 (1.2)	0.496
HSIL/ca (n = 96)	19 (19.8)	3 (3.1)	25 (26.0)	1 (1.0)	39 (40.6)	6 (6.3)	3 (3.1)	
CIN2 (n = 23)	5 (21.7)	0 (0)	4 (17.4)		11 (47.8)	2 (8.7)	1 (4.3)	0.862
CIN3 (n = 45)	10 (22.2)	2 (4.4)	11 (24.4)		18 (40.0)	3 (6.7)	1 (2.2)	

The study participants were divided into community state types (CST’s) based on their bacterial composition and subgrouped according to histopathological diagnosis; Healthy controls (HC), low-grade squamous intra-epithelial lesion (LSIL), high-grade squamous intra-epithelial lesion (HSIL), cervical intra-epitelial neoplasia 2 (CIN2), cervical intra-epitelial neoplasia 3 (CIN3). Four of the CST’s are dominated by *Lactobacill*us species; CST-I (*Lactobaillus crispatus*), CST-II (*Lactobacillus gasseri*), CST-III (*Lactobacillus iners*) and CST-V (*Lactobacillus jensenii*). CST-IV contains mainly non-lactobacilli species, with no dominant species and is subdivided depending on the microbial composition; CST IV-A (high to moderate relative abundance of BVAB1 and *Gardnerella vaginalis*), CST IV-B (high to moderate relative abundance of *Gardnerella vaginalis* and *Fannyhessea vaginae*), CST IV-C (a mixture of aerobic bacteria such as *Prevotella spp, Streptococcus spp, Bifidobacterium spp* and can be further subdivided). *p*-values were determined by chi-square test.

**Figure 4:**
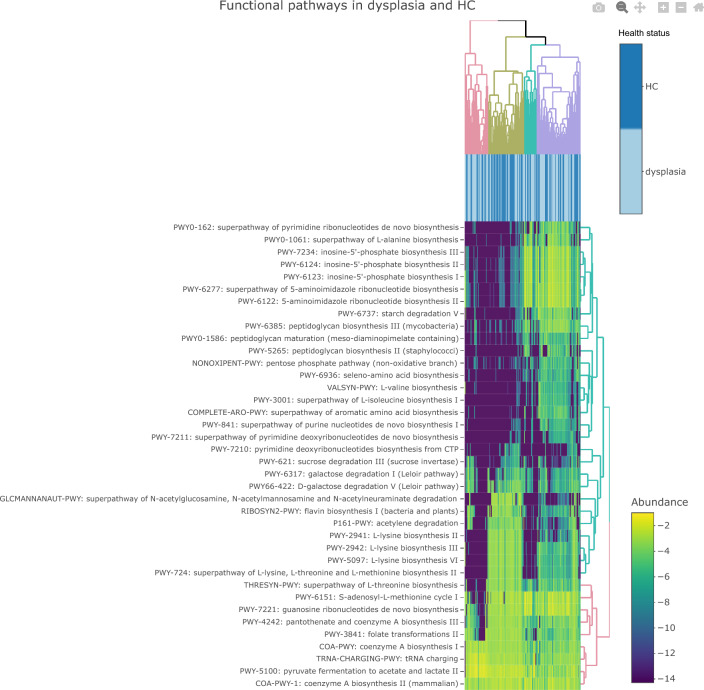
Heatmap of the abundance of functional pathways according to health status of study persons. Heatmap showing the abundance of 38 functional pathways that were significantly differentially abundant between women with dysplasia and healthy controls (HC). Clusters of samples with similar functional properties emerging from the data are highlighted in different colours on top of the heatmap. Functional pathways that clusters due to similar patterns in the data are highlighted in different colours on the right side of the heatmap. Relative abundance data were log2 normalized for better visualization.

To reveal insights into the metabolic potential of the microbial communities in the samples, the metagenomic data was annotated using HUMAnN3 to identify genes present in the samples and classify them into functional pathways. Differential abundance analysis showed that there were 18 pathways with higher abundance in women with dysplasia primarily important for nucleotide biosynthesis and peptidoglycan biosynthesis, and 20 pathways with higher abundance in healthy controls, where several were related to the biosynthesis of amino acids (especially L-lysine) and sugar degradation. (Table S4, Fig. 4).

**Figure 5:**
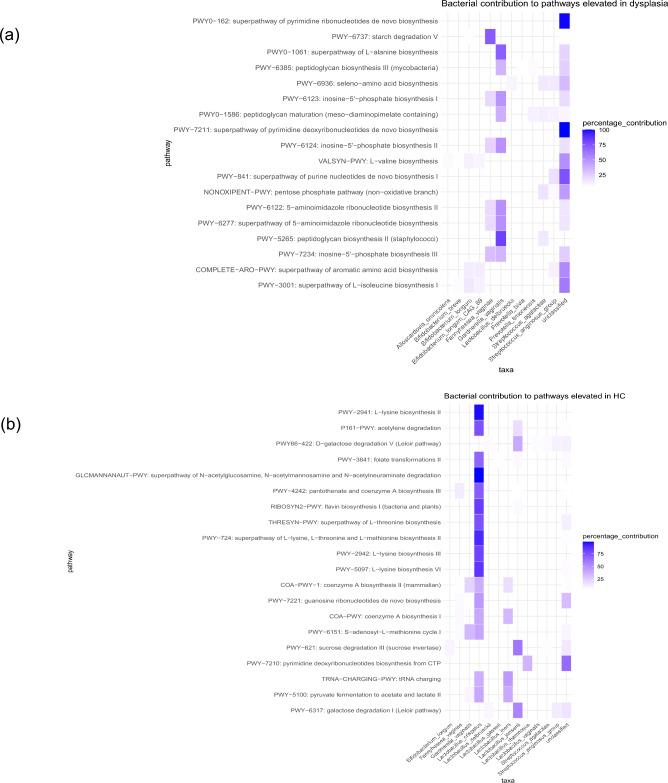
Functional pathways stratified according to contributing species. The major contributing species for each functional pathway with significantly higher abundance in (**a**) women with dysplasia and (**b**) healthy controls (HC). Bacteria that contribute to more than 5% of the total abundance in the cohort for each pathway were included in the visualization.

Furthermore, the community functional profiles were stratified according to contributing species. The analysis revealed that *G. vaginalis* and *F. vaginae* were the predominant bacteria identified, significantly contributing to pathways with higher abundance in women with dysplasia (Fig. 5a). In more detail, *G. vaginalis* was the main bacteria contributing to peptidoglycan biosynthesis and maturation (PWY-5265, PWY-6385, PWY0-1586) and L-alanine biosynthesis (PWY0-1061). Together with *F. vaginae, G. vaginalis* was also contributing to nucleotide biosynthesis through inosine-5'-phosphate biosynthesis (PWY-6123, PWY-6124, PWY-7234) and 5-aminoimidazole ribonucleotide biosynthesis (PWY-6277, PWY-6122). *F. vaginae* was the main bacteria contributing to starch degradation V (PWY-6737). *Streptococcus agalactiae* and anginosus group contributed to some extent to six of the pathways including peptidoglycan biosynthesis and the pentose phosphate pathway (NONOXIPENT-PWY). Pyrimidine biosynthesis pathways (PWY0-162, PWY-7211) could not be associated with a specific bacterium and was assigned unclassified taxa. Unclassified taxa also contributed to many of the other pathways. *Bifidobacterium longum* contributed to some extent to L-valine (VALSYN-PWY), aromatic amino-acid (COMPLETE-ARO-PWY) and L-isoleucine biosynthesis (PWY-3001), together with unclassified taxa.

Pathways with higher abundance in healthy controls were largely dependent on *L. crispatus* and a few pathways relied on L. jensenii (Fig. 5b). *L. crispatus* was the main bacteria contributing to L-lysine biosynthesis (PWY-2941, PWY-2942, PWY-5097), biosynthesis of flavin, L-threonine, L-lysine/L-threonine/L-methionine (RIBOSYN2-PWY, THRESYN-PWY, PWY-724), folate transformation II (PWY-3841) and N-acetyl-glucosamine, -mannosamine and -neruaminate degradation (GLCMANNANAUT-PWY). *L. crispatus* together with *F. vaginae* contributed to pantothenate and coenzyme A biosynthesis III (PWY-4242). *L. crispatus*, *G. vaginalis* and *L. iners* contributed to coenzyme A biosynthesis II (COA-PWY-1). *L. crispatus* and *G. vaginalis* contributed to S-adenosyl-L-methionine cycle I (PWY-6151). *L. crispatus* and *L. iners* contributed to coenzyme A biosynthesis I (COA-PWY), tRNA charging (TRNA-CHARGING-PWY), pyruvate fermentation to acetate and lactate II (PWY-5100). Together with *L. jensenii, L.crispatus* contributed to acetylene degradation (P161-PWY). *L. jensenii* was the main bacteria contributing to galactose and sucrose degradation (PWY66-422, PWY-6317, PWY-621). *L. rhamnosus* and unclassified taxa contributed to pyrimidine DNA biosynthesis from CTP (PWY-7210).

### The microbiome was not associated with different stages of dysplasia

#### Subanalysis comparing LSIL with HSIL/Cancer

First, women with HSIL/Ca (n = 96) were compared with LSIL (n = 81). No significant difference in alpha- or beta diversity, differentially abundant taxa or functional pathways were found when comparing LSIL with HSIL/Ca (Figs. 1 and 2).

For women with LSIL, the most common CST was CST IV (40.7%), followed by CST I (29.6%) and CST III (25%). For women with HSIL/Ca, the most common CST was CST IV (47.9%), followed by CST III (26.0%) and CST I (19.8% %) (Table 2).

#### Subanalysis of women with high grade dysplasia

Women with HSIL were subdivided into moderate HSIL/cervical intra-epithelial neoplasia 2 (CIN2) (n = 23) and cancer in situ/CIN3 (n = 45) for further analysis (Table 1, Table S1). No difference was found between CIN2 and CIN3 in microbiome composition (alpha diversity and beta diversity), taxonomic composition or functional pathways.

The most common CST among women with CIN2 was CST IV (56.5%), followed by CST I (21.7%) and CST III (17.4%). For women with CIN3, the most common CST was CST IV (46.7%), followed by CST III (24.4%) and CST I (22.2%) (Table 2).

#### Vaginal discharge and the microbiota

In the questionnaire, women with HSIL reported more vaginal discharge compared with women with LSIL and it was therefore further investigated if vaginal discharge was associated with the microbiome in these women (Table 1, Table S1). Women with dysplasia (LSIL or HSIL/ca) who reported an abnormal vaginal discharge ((n = 11, mean age 30 (LSIL n = 1, HSIL n = 10)), were compared with women that did not report any abnormal vaginal discharge, and matched for age and stage of dysplasia ((n = 11, mean age 27 (LSIL n = 1, HSIL n = 10)). As women with HSIL/ca also had a higher prevalence of HPV compared with LSIL, HPV status was included in the permanova analysis. Of these 22 women, 17 were positive for high-risk HPV (hrHPV) or potential high-risk HPV (pot hrHPV) and five were positive for low-risk HPV (lrHPV). Vaginal discharge was shown to have a weak but significant association with the microbiome (permanova, *p* = 0.047, R2 = 0.11), while HPV status did not show a significant association with the microbiome.

To investigate what microbial taxa was associated with discharge, differential abundance analysis for the same group of 22 women was performed. The analysis showed higher levels of *P. lacrimalis, A. christensenii, G. vaginalis, Clostridiales* genomosp. BVAB1 and *F. vaginae* in the group of women that reported an abnormal discharge, and higher levels of *Corynebacterium aurimucosum* and *L. jensenii* among women with no reported abnormal discharge (Wilcoxon test *p* < 0.05). No taxa were significant after Benjamini–Hochberg multiple correction.

#### The association between HPV status and the microbiota in women with dysplasia

To investigate if different HPV types were associated with the microbial composition or functional pathways, women were grouped according to presence of hrHPV or pot hrHPV (hrHPV/pot hrHPV), lrHPV or HPV negative. Women with HSIL/ca had a higher prevalence of HPV (all types), HPV16 and hrHPV compared with women with LSIL (Table 3). There was a tendency that women reporting abnormal vaginal discharge also had higher proportions of taxa associated with bacterial vaginosis (BV), with the majority having HSIL and hrHPV positivity. Therefore, these women were excluded from the evaluation of a potential association between the microbiome and HPV.

**Table 3:** HPV-status for the study participants.

	LSIL (n = 81)	HSIL (n = 96)	*p*-value
HPV -status			
HPV negative	17 (21%)	8 (8.3%)	0.00179
Low-risk HPV	17 (21%)	9 (9.4%)	
High- risk HPV or potential high-risk HPV	47 (58%)	79 (82.3%)	
HPV16			
No HPV16	75 (92.6%)	63 (65.6%)	< 0.001
Yes HPV16	6 (7.4%)	33 (34.4%)	

HPV-status according to study participants histopathological diagnosis; low-grade squamous intra-epithelial lesion (LSIL), and high-grade squamous intra-epithelial lesion (HSIL)/cancer.

To account for the different oncogenic risks of the HPV types, comparisons were made with women subdivided into different groups based on HPV type. First, we investigated if being infected with a lrHPV virus had any association to the microbiome composition. Comparing women with lrHPV (n = 24) against HPV negative women (n = 25) we did not see any microbial difference (permanova *p* > 0.05). These groups were thus combined and compared with women infected with a hrHPV or pot hrHPV virus. Women positive for hrHPV or pot hrHPV (n = 49, mean age 37, LSIL n = 34, HSIL n = 15) were compared with women either positive for lrHPV or HPV negative combined as one group (n = 49, mean age 35, LSIL n = 34, HSIL n = 15; lrHPV n = 24 and HPV neg n = 25), and matched for age and stage of dysplasia. Age was included in the permanova (age *p* = 0.138), and analysis showed that positivity for hrHPV or potential hrHPV, when compared with lrHPV and HPV negative, had an association with the microbiota (permanova *p* = 0.025, R2 = 0.024). To follow up this result, differential abundance analysis showed that *F. vaginae*, *P*. *lacrimalis*, *G. vaginalis* and *Mageeibacillus indolicus* were more prevalent in the hrHPV/pot hrHPV group, and that *L. gasseri* was more prevalent in lrHPV/HPV negative group, but none of them passed Benjamini–Hochberg multiple testing correction.

We did not find any specific association between HPV 16 and the microbiota when comparing women positive for HPV 16 with women positive for either other hrHPV types or with HPV negative women.

To investigate if the stage of dysplasia affected the microbiota among women with similar HPV status, additional comparisons were made. Women positive for hrHPV or pot hrHPV were selected (LSIL n = 47, HSIL n = 79), but no difference was found in microbiota diversity attributed to dysplasia stage. There was also no difference found when comparing women positive for lrHPV or HPV negative (LSIL n = 34, HSIL n = 17) attributed to dysplasia stage.

## Discussion

In this study we show that women with cervical dysplasia had increased vaginal microbiota diversity, differentially abundant taxa as well as differences in functional pathways as compared with healthy controls. Our results suggest that the vaginal microbiota may have a role in creating a pro-inflammatory or pro-oncogenic environment in the dysplastic progression from an HPV infection to the subsequent development of cervical cancer.

A higher vaginal microbiota diversity among women with HPV-related disease is well in line with what others have reported^11,18^. Findings on bacterial taxa, also when categorized into CSTs, are similar to previous studies, with a vaginal microbiota consisting mainly of *Lactobacillus* species and *L. crispatus* being more abundant in healthy controls^7,9^. *G. vaginalis, F. vaginae, A. christensenii and P. lacrimalis* were more abundant in women with cervical dysplasia and have all been associated with bacterial vaginosis in previous reports^19,20^. *A. christensenii* has also been associated with chlamydia infection^21^, while *G. vaginalis* have been associated with persistent HPV infection and cervical dysplasia^22,23^. *P. lacrimalis* has furthermore proved to induce an immune response in the host in 3D cervical cancer cell models^13^, and *F. vaginae* has been shown to induce an inflammatory response in 3D cervical cell model^20^.

The vaginal microbiota of women with dysplasia showed higher abundance of 18 functional pathways mainly involved in nucleotide and peptidoglycan biosynthesis. We show that *G. vaginalis* and *F. vaginae* were the main contributing species to these pathways highlighting the close relationship between the two bacteria. *G. vaginalis* was solely driving the peptidoglycan biosynthesis with small contribution from *Streptococcus spp.* Pathways connected to peptidoglycan biosynthesis have previously been associated with cervical cancer^15^. *F. vaginae* were solely responsible for starch degradation (PWY-6737) which has previously been associated with women with bacterial vaginosis^24^. Interestingly *Streptococcus spp*. and *Bifidobacterium spp*. were contributing to several pathways linked to dysplasia, even though these species were not significantly more abundant in women with dysplasia. *Streptococcus spp.* were driving the pentose phosphate pathway (NONOXIPENT-PWY) which has been linked to cancer cell survival and growth and is upregulated in many human cancers^25,26^, as well as being associated with premature birth and a vaginal microbiota depleted of *L crispatus*^27^. *Bifidobacterium longum spp*. contribute to L-valine and aromatic amino acid biosynthesis (VALSYN-PWY, COMPLETE-ARO-PWY). The COMPLETE-ARO-PWY is linked to cancer development through the capability of producing reactive oxygen species (ROS)^28^, and the VALSYN-PWY as well as PWY-6385 have both been associated with gastric cancer tissue^29,30^. Furthermore, the pyrimidine biosynthesis pathway (PWY0-162) is also upregulated in many cancers^31,32^ as well as purine biosynthesis (PWY-841) which can be linked to cancer development through the biosynthesis of purines which reflects the increased energy demands in proliferating cancer cells^33^.

The metabolic landscape associated with dysplasia may indicate an increased demand on metabolic flexibility and competition for nutrients, as well as a focus on growth and proliferation. Further studies are needed to investigate what pathways are actively expressed and how their metabolic output effects the local milieu and thereby the progression from an HPV infection to dysplasia and cancer.

The functional analysis showed 20 pathways with higher abundance in healthy controls, important for amino acid biosynthesis and sugar degradation. *L. crispatus* as the sole species with higher abundance in healthy women, was the main contributing species to these pathways. *L. crispatus* was driving amino acid synthesis foremost L-lysine which has been linked to anti-inflammatory activity^34,35^. *L. jensenii* was an important driver in galactose and sucrose degradation. The S-adenosyl-L-methionine cycle I (PWY-6151) has previously been associated with anti-proliferative, pro-apoptotic and anti-metastatic processes in several types of cancer^36^, and RIBOSYN2-PWY which involves Flavin-dependent enzymes, seems to play a role in cancer prevention^37^. Coenzyme A biosynthesis I (COA-PWY) has also been reported to be more common among healthy women compared to women with bacterial vaginosis^24^, and synthesis of guanosine ribonucleotide (PWY-7221) to be more common among fertile compared with infertile couples^38^., However, a few of the pathways associated with healthy controls in our study have also been linked to cancer, such as TRNA-CHARGING-PWY that has been shown to be deregulated or upregulated in several cancers^39^, and coenzyme A biosynthesis II (COA-PWY-1) that has been associated with cervical cancer^40^. Notably, for both TRNA-CHARGING-PWY and COA-PWY-1 *L. iners* was shown to be one of the major contributing species. *L. iners* has previously been associated with cervical dysplasia, HPV and a less stable vaginal microbiota^9,10^, and its role may require further exploration.

The possible functional properties attributed to the detected differences in pathway abundance suggests that the microbiota may have a role in creating a pro-oncogenic environment in women with dysplasia. Both changes in microbial composition and an HPV-infection may influence the cervicovaginal environment as well as the subsequent tumour microenvironment. Several capabilities of the HPV, including its oncogenes E6 and E7, contribute to the development of cervical cancer through, for example, metabolic reprogramming and other mechanisms affecting the hallmarks of cancer^41,42^. The microbiota may also influence the development of cancer by modulating immune function, or producing metabolites involved in tumour suppression or oncogenesis^43,44^. For a healthy cervico-vaginal environment, several properties of *Lactobacillus* species, such as production of lactic acid have proved beneficial^45^. Conversely, some of the bacteria associated with bacterial vaginosis can produce biofilms and thereby facilitate the HPV infection^22,46–48^, while other bacteria may create a pro-inflammatory environment^13^. These capabilities attributed to different species of the microbiota may result in a synergistic relationship in the microenvironment.

For our sub-analysis comparing LSIL with HSIL, or CIN2 with CIN3, we could not see any significant differences in diversity, taxa or function. However, there was a difference in reported symptoms, when comparing LSIL with HSIL. An abnormal or rich vaginal discharge was more prevalent among women with HSIL as compared with women with LSIL, and showed an association with changes in the microbiota composition. The taxa associated with vaginal discharge, *Peptoniphilus, Aerococcus, Gardnerella,* BVAB1, and *Fannyhessea* have all previously been associated with bacterial vaginosis^19^. Discharge is also one of the Amsel criteria for diagnosing bacterial vaginosis which may further indicate a connection between bacterial vaginosis and cervical dysplasia^49^. Previous studies using 16S marker gene sequencing have reported a stepwise increase in microbiota diversity from healthy controls, across stages of dysplasia, as well as certain species associated with different stages of dysplasia^11,50^. No evident stepwise compositional changes were observed in our metagenomic study, encompassing a larger cohort with histologically confirmed dysplasia. This may be due to limited statistical power or that the microbial changes associated with dysplasia does not differ between the dysplastic stages, but this needs further investigation.

HPV was analysed in relation to dysplasia and microbiota. As expected, we could show that both HPV 16 and other hrHPV were more prevalent among women with HSIL compared with LSIL. HPV 16 was also more prevalent in cancer in situ (CIN 3) compared with moderate HSIL (CIN 2). This is well in line with what epidemiological studies have shown^51^. Our results show that low-risk HPV is not associated with a difference in microbiota, while women infected with a high-risk or potential high-risk HPV show an altered microbiota. There was a trend that this effect is driven by higher abundance of *F. vaginae, P. lacrimalis, G. vaginalis,* and *M. indolicus*, and lower levels of *L. gasseri,* which is similar to previous findings^13,20, 22^. Previous reports also associate *L. gasseri* with remission of HPV infection^10^. When looking specifically at HPV 16 no specific association to the microbiome was found. These results indicate a probable association between the vaginal microbiota and HPV, but to understand how this association may apply to specific HPV types, larger groups infected with each subtype is required.

A major strength of this study is the detailed classification of dysplasia and healthy controls, where the dysplasia was histologically verified, as opposed to only cytology, and the healthy controls were verified with two consecutive normal screening test and no HPV positivity or abnormal cervical histo- or cytopathological diagnosis during the full duration of the study period. Another strength is that this study is one of the first large metagenomic sequencing studies on the vaginal microbiota and cervical dysplasia, presenting findings on both microbial composition and functional properties. Furthermore, as there are few studies of this size, from this region in the world, the findings contribute to the understanding on how the vaginal microbiota may vary between different parts of the world.

Study limitations include the question of causality, whether the HPV infection induces a change in the microbiota or if changes in the microbiota facilitate an HPV infection. However, both longitudinal and mechanistic studies suggest that the microbiota could have an effect on HPV related disease^12,13, 52^. There may be a reciprocal interaction between multiple components of the microenvironment, such as microbiota and different parts of the immune system. Furthermore, the interpretation of the analysis of women with discharge should be made with caution, as the vaginal discharge was a self-reported symptom and is therefore highly subjective.

From a clinical perspective, it is possible that a certain composition of the vaginal microbiota or bacterial taxa, in combination with a specific HPV-type or stage of cervical dysplasia, could require new modalities for surveillance or treatment. A potential future therapeutic perspective includes the possibility to modulate the vaginal microbiota, in order to prevent or treat HPV related disease^53,54^. A better understanding of carcinogenesis may enable earlier detection and treatment, before progression to more advanced stages of dysplasia and cancer.

In summary, women with cervical dysplasia had higher microbial diversity, and differences in bacterial taxa and functional pathways as compared with healthy controls. This study is, to our knowledge, one of the largest metagenomic studies on cervical dysplasia, HPV and microbiota. It confirms previous findings on microbial composition and adds new knowledge on which bacteria and pathways might be involved in the pathogenesis of dysplasia. These findings further support the hypothesis that the cervicovaginal microbiota may have an active role in the cervical carcinogenesis. Interactions between the microbiota, HPV and other possible components of the tumour microenvironment therefore require further exploration.

## Materials and methods

### Study population and sampling

Study participants were recruited between June 2017 and January 2020. Women with histologically verified dysplasia and cancer (n = 177; low grade dysplasia (LSIL) n = 81, high-grade dysplasia (HSIL) n = 94, cancer n = 2) were recruited at the gynaecological out-patient clinic at Uppsala University hospital, Sweden. Healthy controls were recruited by sending invitations by regular mail to all women invited to the organized cervical screening programme in Uppsala County, Sweden. Women with two consecutive normal screening tests from the cervical screening programme, and no abnormal cervical pathology, including histology, cytology and HPV, during the full duration of the study period served as healthy controls (n = 177). Women with dysplasia were matched against the healthy controls based on age using the R package MatchIt v. 4.5.3^55^, with one control per case, using nearest neighbour method. For women with multiple samples, only one sample was used per woman. Known pregnant or breastfeeding women were excluded.

A short questionnaire was completed by the clinical staff at the time of the gynaecological examination and sampling, focusing on current gynaecological symptoms (Table 1, Table S1).

Vaginal microbiota samples were collected by gynaecologists at the gynaecological out-patient clinic, Uppsala University Hospital, and for the controls by midwives at maternity care centres in Uppsala county. Samples were collected with vaginal swabs (FLOQSwabs™, Copan Flock Technologies, Brescia, Italy) during gynaecological examination. Swabs were then transferred into FluidX tubes (Brooks Life Sciences, Chelmsford, MA, USA) containing 0.8 ml DNA/RNA-shield (Zymo Research, Irvine, CA, USA) as previously described^56^.

All cytological and histological analyses, as well as HPV analysis with HPVir assay^57^, were performed at the department of Clinical Pathology and Cytology, Uppsala University Hospital, Uppsala, Sweden.

All participation was voluntary, and participants provided written informed consent. The study was approved by Uppsala Regional Ethics Committee Dnr 2016/517. The study was performed in accordance with approved guidelines including the Declaration of Helsinki.

### DNA extraction and sequencing

DNA was extracted as previously reported^56^. Briefly, DNA was quantified with Quant-iT dsDNA Assay kits (Invitrogen, ThermoFisher, USA), and normalized to 50ng DNA. MGIEasy FS DNA Library Prep Set kit (MGI, Shenzhen, China) was used to prepare DNA libraries. Quality control of all libraries was done by measuring their concentration with Quant-iT dsDNA Assay kits and fragment size with TapeStation D1000 kit (Agilent, USA). Libraries were pooled with an equal amount of DNA from each sample. Circularized DNA libraries were sequenced using 100 bp paired-end sequencing on a DNBSEQ-T7 sequencer (MGI, Shenzhen, China). Positive (ZymoBIOMICS Community standard, Zymo Research, Irvine, CA, USA, Supplementary Table 6) and negative (DNA/RNA shield) controls were included for both extraction and sequencing steps.

### Taxonomic and functional annotation

Shotgun libraries were analyzed and annotated using the Snakemake workflow (ctmrbio/stag-mwc: StaG v0.5.0). First, human DNA was identified by mapping against the human genome version GRCh38 using Kraken2 (v2.0.8-beta)^58^, and after removal of human DNA, taxonomic annotation was performed using Kraken2 (v2.0.8-beta) mapping to the vaginal bacteria database OptiVag DB v3 supplemented with complete NCBI RefSeq Fungi database^58^, and Bracken correction as previously described^56^. Functional annotation was performed using metaphlan3^59^ and humann3^59^. Samples not attaining 20 M total reads or 0.1 M annotated microbial reads were excluded.

For taxonomic analysis any species detected by < 500 reads were set to zero to reduce noise. Decontam R package (v1.18.0) with batch function (threshold 0.1) was used to identify possible contaminant taxa and 26 taxa were identified and removed from the analysis^60^ (Table S5).

To remove very low abundant taxa or functional pathways, only features with more than 0.005% relative abundance in at least 10 samples were included in the analysis.

### Statistical analyses

All statistical analyses were performed in R (v4.2.2). The descriptive table statistics was performed using Kruskal–Wallis and Chi-Square test with R package Table1 (v1.4.3) (https://github.com/benjaminrich/table1), and graphs were generated with packages RColorBrewer (v1.1–3), Vioplot (v0.2) and Pheatmap (v1.0.10).

### Diversity measures

Alpha-diversity was calculated on filtered relative abundance values using Shannon, Inverse Simpson and species number using the R package vegan (v2.6.4)^61^, with pairwise Wilcoxon test for significance testing between groups. Beta-diversity differences were calculated with PERMANOVA analysis (adonis2 from vegan package) on center-log-ratio (CLR) transformed and filtered feature values using Euclidian distance and 999 permutations.

### Differential abundance analysis

To identify differences in the abundances of individual taxa or functions between groups the ALDEx2 R package (v1.24.0) was used^62,63^. This method estimates technical variation within each sample per taxon by utilizing the Dirichlet distribution. Filtered counts were used as input, 128 Monte Carlo samples were used to estimate the underlying Dirichlet distribution, and values were converted to centered log-ratio transform. False discovery rate adjusted (Benjamini-Hochberg) Wilcoxon Rank-Sum test *p*-values < 0.05 were considered significant. ALDEx2 also calculates expected standardized effect sizes by incorporating the variation within groups and between groups.

### Classification of vaginal samples into community state types

The vaginal samples were classified into community state types (CST) with Valencia as described^17^. CSTs were introduced to create a common classification system of vaginal microbiome samples based on their bacterial composition. CST-I (dominated by *L. crispatus*), CST-II (dominated by *L. gasseri*) and CST-V (dominated by *L. jensenii*) are considered the most beneficial CSTs, while CST-III (dominated by *L. iners*) more easily shift between a Lactobacilli-dominated to a high diverse composition, the latter defined as CST-IV (mainly non-lactobacilli species, with no dominant species). These CSTs can also be sub grouped further depending on the composition^17^.

### HPV analysis

A subset of samples (n = 49) had available HPV typing results from the clinic, using the HPVir assay^61^. To assess the remaining samples data from a 27-plex Luminex^64^ was used (n = 216) as well as metagenomically typed HPV using the HPViewer tool (n = 354)^65^. To investigate associations between HPV and microbiome composition in our study a sample was considered positive for a HPV type if any of the three assays showed a positive result.

The HPV types were divided into low-risk (lrHPV); HPV6, HPV11, HPV42, HPV43, HPV44, HPV70, potential high-risk (pot hrHPV); HPV26, HPV30, HPV53, HPV66, HPV67, HPV69 and high risk (hrHPV); HPV16, HPV18, HPV31, HPV33, HPV35, HPV39, HPV45, HPV51, HPV52, HPV56, HPV58, HPV59, HPV68, HPV73, HPV82^66,67^.

With HPViewer additional HPV types were detected that has previously been reported as either low risk or undetermined, and was treated as lrHPV in our analysis; HPV91, HPV40, HPV62, HPV84, HPV87, HPV90, HPV106, HPV114, HPV61, HPV89, HPV121, HPV54, HPV103, HPV83, HPV32, HPV101, HPV74, HPV129, HPV108, HPV81, HPV98, HPV34, HPV71, HPV155, HPV80, HPV107, HPV72, HPV37, HPV168, HPV119, HPV149, HPV163, HPV124, HPV118, HPV115^66–68^.

### Supplementary Information

Supplementary Table 1.

Supplementary Table 2.

## Data Availability

The raw sequence data generated in this study have been deposited in the European Nucleotide Archive (ENA) under the accession numbers PRJEB72778, PRJEB72779. Metadata is available in Supplementary Table 7.
