# Supplementary material for: Compositional and functional differences of the vaginal microbiota of women with and without cervical dysplasia
Source: Sci Rep. 2024 May 16;14:11183. doi: 10.1038/s41598-024-61942-2 (PMC11099171; doi:10.1038/s41598-024-61942-2)
Supplement: Supplementary file 2 — Supplementary Table 2. [file 41598_2024_61942_MOESM2_ESM.pdf]

Compositional and functional differences of the vaginal microbiota of women with and without cervical dysplasia

Authors and affiliations: Johanna Norenhag[1#*], Gabriella Edfeldt[2#], Karin Stålberg[1], Fabricio Garcia[2], Luisa Warchavchik Hugerth [3], Lars Engstrand[2], Emma Fransson[1,2], Juan Du[2], Ina Schuppe-Koistinen[2], Matts Olovsson[1]

1 Department of Women's and Children's Health, Uppsala University, Uppsala, Sweden

2 Centre for Translational Microbiome Research, Department of Microbiology, Tumor and Cell Biology (MTC), Karolinska Institutet, Stockholm, Sweden

3 Department of Medical Biochemistry and Microbiology, Science for Life Laboratory, Uppsala University, Uppsala, Sweden

# Contributed equally

* Corresponding author: Johanna Norenhag, Johanna.norenhag@kbh.uu.se

Supplementary Table 1. Gynaecological symptoms of the study participants. Clinical information and gynaecological symptoms from questionnaires with study participants grouped according to histopathological diagnosis; Healthy controls (HC), dysplasia of any type (Dysplasia), low-grade squamous intra-epithelial lesion (LSIL), high-grade squamous intra-epithelial lesion (HSIL), cervical intra-epithelial neoplasia 2 (CIN2), cervical intra-epithelial neoplasia 3 (CIN3). P-values were calculated using Kruskal-Wallis or Chi-Square test.

	**HC (n=177)**	**Dysplasia (=177)**	**p-value**	**LSIL (n=81)**	**HSIL/ca (n=96)**	**p-value**	**CIN2 (n=23)**	**CIN3 (n=45)**	**p-value**
**Gynaecological symptoms <24 hours from sampling**			0.0558			1			0.353
Yes	21 (11.9%)	35 (19.8%)		16 (19.8%)	19 (19.8%)		6 (26.1%)	6 (13.3%)	
No	139 (78.5%)	125 (70.6%)		57 (70.4%)	68 (70.8%)		15 (65.2%)	34 (75.6%)	
Missing	17 (9.6%)	17 (9.6%)		8 (9.9%)	9 (9.4%)		2 (8.7%)	5 (11.1%)	
**Spotting**			0.216			0.842			0.741
Yes	1 (0.6%)	5 (2.8%)		3 (3.7%)	2 (2.1%)		1 (4.3%)	0	
No	159 (89.8%)	157 (88.7%)		70 (86.4%)	85 (88.5%)		20 (87%)	40 (88.9%)	
Missing	17 (9.6%)	17 (9.6%)		8 (9.9%)	9 (9.4%)		2 (8.7%)	5 (11.1%)	
**Odour**			0.615			0.309			1
Yes	1 (0.6%)	3 (1.7%)		0	3 (3.1%)		0	1 (2.2%)	
No	159 (89.8%)	157 (88.7%)		73 (90.1%)	84 (87.5%)		21 (91.3%)	39 (86.7%)	
Missing	17 (9.6%)	17 (9.6%)		8 (9.9%)	9 (9.4%)		2 (8.7%)	5 (11.1%)	
**Irritation, itching**			0.172			0.967			0.56
Yes	4 (2.3%)	10 (5.6%)		4 (4.9%)	6 (6.3%)		2 (8.7%)	1 (2.2%)	
No	156 (88.1%)	150 (84.7%)		69 (85.2%)	81 (84.4%)		19 (82.6%)	39 (86.7%)	
Missing	17 (9.6%)	17 (9.6%)		8 (9.9%)	9 (9.4%)		2 (8.7%)	5 (11.1%)	
**Pain**			0.176			0.189			0.894
Yes	2 (1.1%)	7 (4%)		1 (1.2%)	6 (6.3%)		2 (8.7%)	2 (4.4%)	
No	158 (89.3%)	153 (86.4%)		72 (88.9%)	81 (84.4%)		19 (82.6%)	38 (84.4%)	
Missing	17 (9.6%)	17 (9,6%)		8 (9.9%)	9 (9.4%)		2 (8.7%)	5 (11.1%)	
**Vaginal discharge with blood**			0.0734			0.812			1
Yes	1 (0.6%)	7 (4%)		4 (4.9%)	3 (3.1%)		0	1 (2.2%)	
No	159 (89.8%)	153 (86.4%)		69 (85.2%)	84 (87.5%)		21 (91.3%)	39 (86.7%)	
Missing	17 (9.6%)	17 (9.6%)		8 (9.9%)	9 (9.4%)		2 (8.7%)	5 (11.1%)	

Supplementary Table 2. Differential abundance of taxa comparing women with dysplasia with healthy controls (dataset in separate .xlsx file).

Supplementary Table 3. Microbial compositions based on community state types (CST's) according to study participant's histopathological diagnosis

The study participants were divided into community state types (CST's) based on their bacterial composition and subgrouped according to histopathological diagnosis; Healthy controls (HC), low-grade squamous intra-epithelial lesion (LSIL), high-grade squamous intra-epithelial lesion (HSIL), cervical intra-epitelial neoplasia 2 (CIN2), cervical intra-epitelial neoplasia 3 (CIN3). Four of the CST's are dominated by Lactobacillus species; CST-I (*Lactobaillus crispatus*), CST-II (*Lactobacillus gasseri*), CST-III (*Lactobacillus iners*) and CST-V (*Lactobacillus jensenii*). CST I can be subdivided into CST I-A with contains almost completely *L. crispatus*, and CST I-B with less *L. crispatus*, but still in majority. Similarily CST III include subtypes CST III-A which contains almost only *L. iners*, and CST III-B less *L. iners* but still majority. CST-IV contains mainly non-lactobacilli species, with no dominant species and is subdivided depending on the microbial composition; CST IV-A (high to moderate relative abundance of BVAB1 and *Gardnerella vaginalis*), CST IV-B (high to moderate relative abundance of *G. vaginalis* and *Fannyhessea vaginae*) and CST IV-C which contains a low relative abundance of *G. vaginalis*, BVAB1 and *lactobacilli spp*, and can be further subdivided as follows; CST IV-C0 contains a relatively even community with *Prevotella spp*., CST IV-C1 is dominated by *Streptococcus spp*., and CST IV-C3 which is dominated by *Bifidobacterium spp*. P-values were determined by chi-square test.

Number of study participants n, (%)	CST I-A	CST I-B	CST II	CST III-A	CST III-B	CST IV-A	CST IV-B	CST IV-C0	CST IV-C1	CST IV-C3	CST V	p-value
HC (n=177)	56 (31.6)	19 (10.7)	4 (2.3)	19 (10.7)	17 (9.6)	2 (1.1)	42 (23.7)	6 (3.4)	3 (1.7)	2 (1.1)	7 (4.0)	0.0122
Dysplasia (n=177)	28 (15.8)	15 (8.5)	5 (2.8)	24 (13.6)	22 (12.4)	2 (1.1)	70 (39.5)	2 (1.1)	1 (0.6)	4 (2.3)	4 (2.3)	
LSIL (n=81)	15 (18.5)	9 (11.1)	2 (2.5)	14 (17.3)	7 (8.6)	1 (1.2)	31 (38.3)	0 (0)	1 (1.2)	0 (0)	1 (1.2)	0.269
HSIL/ca (n=96)	13 (13.5)	6 (6.3)	3 (3.1)	10 (10.4)	15 (15.6)	1 (1.0)	39 (40.6)	2 (2.1)	0 (0)	4 (4.2)	3 (3.1)	
CIN2 (n=23)	4 (17.4)	1 (4.3)	0 (0)	2 (8.7)	2 (8.7)	0 (0)	11 (47.8)	1 (4.3)	0 (0)	1 (4.3)	1 (4.3)	0.945
CIN3 (n=45)	7 (15.6)	3 (6.7)	2 (4.4)	3 (6.7)	8 (17.8)	0 (0)	18 (40.0)	1 (2.2)	0(0)	2 (4.4)	1 (2.2)	

Supplementary table 4. Functional Pathways. Differential abundance analysis of functional pathways for women with dysplasia and healthy controls (dataset in separate .xlsx file).

Supplementary table 5. Contaminant taxa identified and removed from the analysis. The listed 26 taxa were identified as contaminant taxa and removed from the analysis (dataset in separate .xlsx file).

Supplementary table 6. Positive controls. Taxonomic annotation for the positive controls (dataset in separate .xlsx file).

Supplementary table 7. Metadata. Metadata and sampleID that match the ENA files (dataset in separate .xlsx file).
